# p-Coumaric acid protects cardiac function against lipopolysaccharide-induced acute lung injury by attenuation of oxidative stress

**DOI:** 10.22038/ijbms.2019.36316.8650

**Published:** 2019-08

**Authors:** Maryam Kheiry, Mahin Dianat, Mohammad Badavi, Seyyed Ali Mard, Vahid Bayati

**Affiliations:** 1 Department of Physiology, Physiology Research Center, Faculty of Medicine, Ahvaz Jundishapur University of Medical Sciences, Ahvaz, Iran; 2 Cellular and Molecular Research Center, Faculty of Medicine, Ahvaz Jundishapur University of Medical Sciences, Ahvaz, Iran

**Keywords:** Acute lung injury, ECG, Hemodynamic parameters, LPS, Nrf2, p-Coumaric acid

## Abstract

**Objective(s):**

Acute lung injury (ALI) has a high mortality rate and is characterized by damage to pulmonary system giving rise to symptoms such as histological alteration, lung tissue edema and production of proinflammatory cytokine. p-Coumaric acid (p-CA), as a phenolic compound, that is found in many types of fruits and vegetables has been reported to exhibit a therapeutic effect in several inflammatory disorders. The aim of our study was evaluation of pretreatment with p-CA against heart dysfunction, oxidative stress and nuclear factor-erythroid 2 -related factor 2 (Nrf2) modifications following lipopolysaccharide (LPS)-induced acute lung inflammation.

**Materials and Methods:**

The rats were divided into four groups (n=8): Control, LPS (5 mg/kg, it), p-CA (100 mg/kg, IP), and LPS+pCA. Inflammatory response and oxidative stress were evaluated by measurement of interleukin 6 (IL-6), tumor necrosis factor alpha (TNF-α) and malondialdehyde (MDA) levels in heart tissue. For evaluation of the effect of LPS on cardiac response, electrocardiography (ECG) and hemodynamic parameters were recorded.

**Results:**

A significant increase in lipid peroxidation (*P*<0.001, cytokine parameters (TNF-α and IL-6 (*P*<0.01), gene expression of Nrf2 (*P*<0.05), and antioxidant activity of superoxide dismutase and glutathione (*P*< 0.05) in addition to glutathione peroxidase (*P*<0.01) was demonstrated in heart tissue of ALI rats. LPS can impair cardiac function (in *in vitro *measurement of hemodynamic parameters by using Langendorff setup, and in* in vivo *measurement of ECG parameters), and pretreatment with p-CA recovered these parameters to control levels in heart. Pretreatment with p-CA causes modulation of cytokines and MDA level that protected cardiac injury caused by LPS in ALI model.

**Conclusion:**

Our results showed anti-inflammatory and antioxidative effect of p-CA on LPS-induced ALI.

## Introduction

Acute lung injury (ALI) is related to inflammation in pulmonary system and leads to serious illness and death (1). In lipopolysaccharide (LPS)-induced inflammation, alveolar–capillary barrier is disrupted, and lung permeability is increased, which leads to infiltration of neutrophils into the lungs (2). Chronic and acute lung injury has a profound effect on cardiovascular system. Many studies have shown that airway exposures to cigarette smoke, pollutants and infectious agent leads to cardiac diseases (3). Evidences show association between lung and cardiac disease (4). Cardiac disorders associated with lung inflammation increase morbidity and morbidity. Patients with lung disease also show an increased risk of mortality due to heart failure, myocardial infarction and arrhythmia compared to healthy individuals. Since the cardiac dysfunction and abnormalities obviously contribute to the overall morbidity associated with pulmonary disease (5); therefore, an understanding of their role and potential for treatment is necessary.

 Also, ALI has been reported to lead to systemic inflammation and increased endothelial dysfunction in systemic blood vessels and disrupted cardiac output, which are major risk factors for the cardiovascular system (6, 7). The concentration of interleukin 6 (IL-6), as a proinflammatory cytokine, increases in bronchoalveolar lavage fluid (BALF), and in the lung upon exposure to particulate matter (8), but its high concentration in the blood poses a cardiovascular risk factor in patients with coronary artery disease (9). LPS initiates a sequence of cellular disorders, which reduce cardiac contractile efficiency (10). Systemic infections lead to serious destruction in cardiomyocytes, such as cell apoptosis, impairment of calcium homeostasis and excitation/contraction coupling (11). 

Recently, several plant-derived compounds have been found to be immunosuppressive, and are now used as an anti-autoimmune and anti-inflammatory factor (12). p-Coumaric acid (p-CA) is a phenolic compound, which is found in vegetables, fruits, and other herbal products (cranberry syrups, rice, grape juices, tomatoes, and apple) (13). p-coumaric acid can convert to phenolic acids such as chlorogenic acid, rosmarinic acid, flavonoids, and other secondary metabolites and also possesses various effects including antioxidant, anti-angiogenic, anti-UV damage, and anti-platelet properties (14). Nuclear factor-erythroid 2 -related factor 2 (Nrf2), a transcription factor, binds to antioxidant response elements encoding antioxidant enzymes such as glutathione S-transferase (GST), NAD(P)H dehydrogenase quinone 1 (NQO1), heme oxygenase-1, glutathione peroxidase (GPx), NAD(P)H quinone oxidoreductase, and glutamate cysteine ligase (GCL) (15). Via scavenging the cytotoxic electrophile agents and reactive oxygen species (ROS), and responding to pro-inflammatory stimuli, it plays a key role in cellular defense (16).

In this study, we used LPS to induce ALI. We hypothesized that systemic inflammation during ALI induces myocardial dysfunction through oxidative stress. Therefore, we investigated the *in vitro* and *in vivo* effects of p-CA in heart injury followed by LPS-induced ALI.

## Materials and Methods

LPS (*Escherichia coli* LPS, 055:B5), p-CA (Sigma-Aldrich, USA), xylazine 2%, ketamine HCl 10% (Alfasan Co. Netherlands), antioxidant assay kits (ZELLBIO, Germany), Krebs salts (Merck, Germany), and ELISA kits (IBL, Germany) were provided.

Thirty two young male rats (Sprague-Dawley, weighting 180–200 g) were purchased from animal house of Ahvaz Jundishapur University of Medical Sciences, Ahvaz, Iran. The animals were divided into four groups: Control, pCA, LPS, and LPS+pCA (n=8): Rats received saline (Control) or p-CA (100 mg/kg) intraperitonealy for a period of ten days prior to the intratracheal (IT) administration of saline on the 8^th^ day. LPS (5 mg/kg, IT) was instilled in the airway (17) on the 8^th^ day in LPS and LPS+ pCA groups. Rats received saline or pCA (100 mg/kg) (18) intraperitonealy for a period of ten days prior to the intratracheal administration of LPS on the 8^th^ day. The rats were sacrificed 72 hr after LPS or saline treatment. Concentration-effect study (25, 50 and 100 mg/kg, IP) was performed with p-CA to determine the effective dose. In heart tissue, p-CA 25 mg/kg does not have significant effect on tumor necrosis factor alpha (TNF-α), but p-CA 50 (*P*<0.05) and 100 mg/kg can (*P*<0.001) decreased TNF-α level, and p-CA at 100 mg/kg can significantly inhibit TNF-α as marker of inflammation. The experiments were carried out in accordance with the ethical guidelines, and the protocol was approved by the Ethics Committee for Animals at Ahvaz Jundishapur University of Medical Sciences, Ahvaz, Iran (No: IR.AJUMS.REC.1396.275).

***LPS instillation***

After anesthetizing the animals by Xylazine and Ketamine (IP), the normal saline containing 5 mg/kg of *Escherichia coli* lipopolysaccharide was instilled into the airways. Control animals received saline by the same route (17).

***Cardiac TNF-α and IL-6 analyses***

The rats were anesthetized and sacrificed 72 hr after injection of LPS or normal saline. Then, 100 mg of heart tissue was used to homogenize and centrifuged at 4000 rpm. The supernatant was kept at -80 ºC for other analyses. IBL kits (Germany) was used for determination of the TNF-α and IL-6 levels.

***Evaluation of electrocardiography***

Seventy-two hr after LPS or saline treatment, the animals were anesthetized, and cardiac function was examined 72 hr after LPS administration (Powerlab, ADInstruments, Australia) in all groups for 15 min**. **The electrodes were connected to a Bioamplifier and digitalized using an A/D converter, Powerlab 8sp. Then, the heart rate (HR), PR, QT, RR, QRS interval, and the QRS complex voltage were measured using Chart software (ADInstruments, Australia). By using Bazett’s formula (QTc = QT interval/square root of the RR interval), the corrected QT interval (QTc) was calculated (19).

***Preparation of isolated hearts*** ***using Langendorff*** ***setup***

The trachea was cannulated after anesthesia, and then ventilation was performed using a rodent ventilator (UGO BASILE Co., model 7025). The aorta was cannulated and heart was removed from the animal’s body, severing the blood vessels; transmitted to a Langendorff setup, it was then perfused in a reverse fashion via the aorta with a nutrient rich, oxygenated solution (Krebs–Henseleit solution at temperature of 37± 0.1 °C and a constant flow of 10 ml/min). To allow stabilization of coronary perfusion pressure, the hearts were perfused for 30 min. The balloon volume was set to maintain a left ventricular end diastolic pressure (LVEDP) of 5 mmHg. The signal from the pressure transducer was analyzed using a PowerLab system (ADInstruments, Australia). Indicator of hemodynamic status such as left ventricular end systolic pressure (LVESP), HR, LVEDP, perfusion pressure, left ventricular developed pressure (LVDP: LVSP-LVEDP), ±dp/dt: Maximal and minimum rate of pressure development and rate pressure product (calculated as HR × LVDP) were measured. HR and perfusion pressure were continuously monitored (20).

***Antioxidant enzymes and lipid peroxidation***

After treatment of all groups, we homogenized 100 mg of heart tissue in 1 ml of PBS (50 mM at pH 7.4) and then centrifuged (4000 rpm, 10 min). For measurement of superoxide dismutase (SOD), GPx, and glutathione (GSH) activities, and malondialdehyde (MDA) levels (ZellBio GmbH kits, Germany), the supernatant was collected and analyzed. 

***Expression of Nrf2 gene***

RNeasy plus mini kit (Qiagen Co, Netherlands) was used for RNA extraction. The total RNA was extracted from the homogenized tissue and purity of the total RNA was measured by spectrophotometry at 260 and 280 nm (BioPhotometer Plus; Eppendorf, Germany). One μg of total RNA was used for complementary DNA (cDNA) synthesis (cDNA synthesis kit (Qiagen USA). A light cycler PCR (Roche, Diagnostics) was used to determine the levels of Nrf2 mRNA and the housekeeping gene glyceraldehyde-3-phosphate dehydrogenase (GAPDH). Sequences of our primers (Bioneer, Daejeon, South Korea) were: Nrf2 (Forward: 5’ GGTTTCTTCGGCTACGTTTC 3’ and reverse: 5’ CCTCCCAAACTTGCTCAATG 3’), GAPDH (Forward: 5’ GTATTGGGCGCCTGGTCACC 3’ and reverse: 5’ CGCTCCTGGAAGATGGTGATGG 3’) (21).

***Statistical analysis***

Statistical analyses were performed and described as means *± *SEM. Data comparisons were made by the Student’s t-test or one-way analysis of variance followed by the Tukey-Kramer multiple comparisons test. Results were considered significant if *P*<0.05.

## Results

***Confirmation of LPS-induced systemic inflammation***

**Figure 1:**
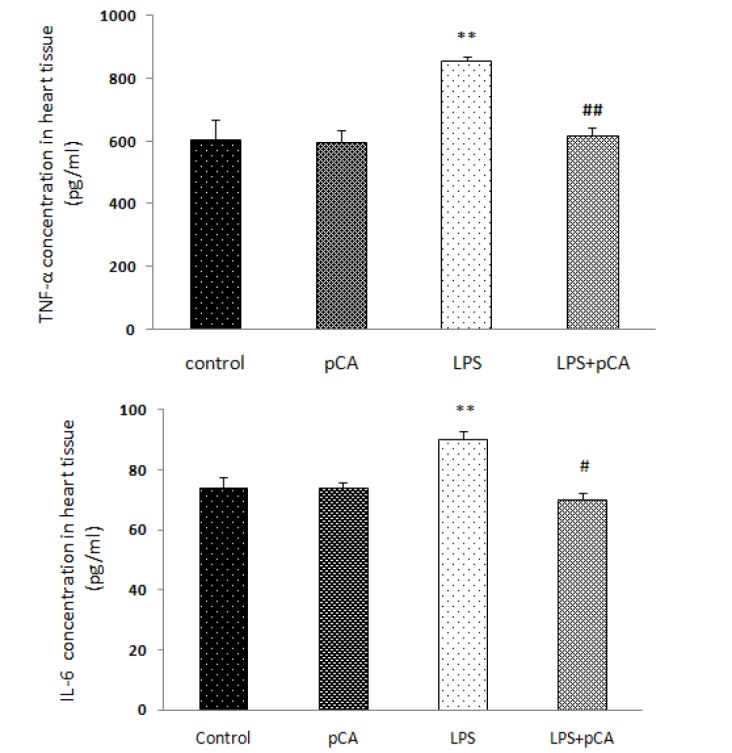
Effects of p-CA on LPS-induced systemic inflammation. The concentrations of TNF-α and IL-6 in heart tissue analyzed by ELISA. Data are expressed as the mean±SEM (n=8)

IL-6 and TNF-α levels were analyzed 72 hr after LPS exposure, which were significantly higher compared to control group (*P*<0.01, Figure 1) suggesting that the LPS induced systemic inflammation.

***Effects of p-CA on antioxidant enzymes’ activity and Lipid peroxidation***

**Figure 2:**
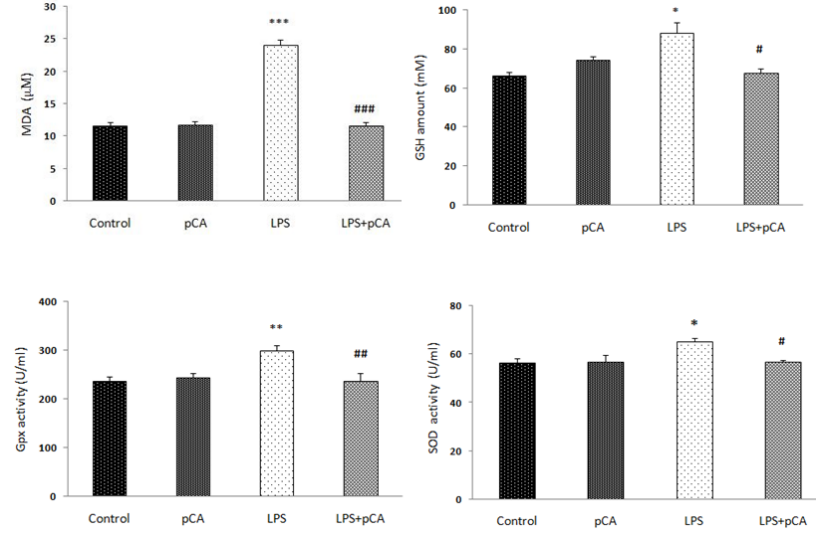
Effects of p-CA on LPS-induced systemic inflammation. The concentrations of antioxidant enzymes and MDA levels in heart tissue analyzed by ZellBio kits. Data are expressed as the mean±SEM (n=8). * *P*<0.05, ** *P*< 0.01, *** *P*<0.001 versus the control group; # *P*<0.05, ## *P*<0.01, ### *P*<0.01 versus the LPS group. LPS: Lipopolysaccharide; p-CA: p-Coumaric acid; MDA: Malondialdehyde

The effect of LPS was investigated on antioxidant enzymes’ activity and lipid peroxidation in heart tissue. As shown in Figure 2, SOD (*P*<0.05), GPx (*P*<0.01), GSH (*P*<0.05), and MDA (*P*<0.001) levels increased significantly in LPS (5 mg/kg) group compared to control group. In groups receiving p-CA, a significant decrease was found in these antioxidant enzymes and MDA level compared to LPS rats. 

***ECG measurements***

As shown in Table 1, there was an increase in HR (bpm) (*P*<0.01), and RR interval (S) (*P*<0.01), QRS Complex (S) (*P*<0.01) and PR interval (S) (*P*<0.01), and QT interval (S) (*P*<0.01), while QRS complex (mv) (*P*<0.01) decreased in LPS rat compared to control group. Also, in group that received p-CA, these alterations significantly restored compared to LPS group.

***Hemodynamic measurements***

Hemodynamic results are shown in Table 2. There was a decrease in +dp/dt (mmHg) (*P*<0.01), rate pressure product (RPP) (*P*<0.001), LVDP (mmHg) (*P*<0.05), and LVEDP (mmHg) (*P*<0.05), whereas HR (*P*<0.001) increased in LPS compared to control group, but pretreatment with p-CA blocked the induction of these effects. However, no effect was observed on other parameters, such as LVSP (mmHg), dp/dt min (mmHg), and perfusion pressure (mmHg).

***Effects of p-CA on Expression of Nrf2 gene***

**Figure 3:**
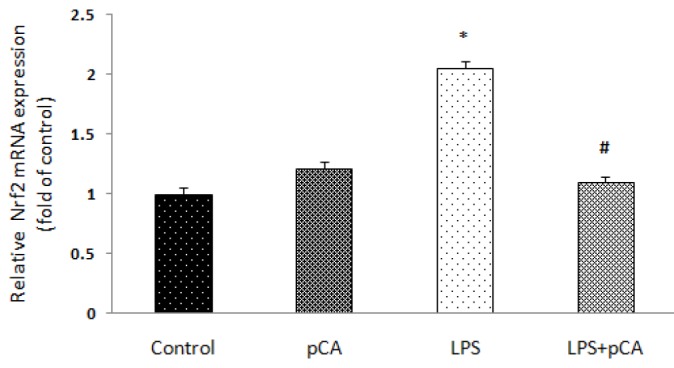
Effects of p-CA on LPS-induced systemic inflammation. Nrf2 mRNA expression in heart tissue analyzed. Data are expressed as the mean±SEM (n=8). * *P*<0.05, versus the Control rats. # *P*<0.05 versus the LPS group. Nrf2: Nuclear factor-erythroid 2 -related factor 2; LPS: Lipopolysaccharide; p-CA: p-Coumaric acid

As presented in Figure 3, Nrf2 mRNA expression significantly increased 72 hr after LPS exposure in comparison with controls (*P*<0.05). However, p-CA pretreatment caused significantly decreased in mRNA expression of Nrf2 compared to LPS groups (*P*<0.05).

## Discussion

Present findings with an *E. coli* induced ALI model indicate that intratracheal administration of bacterial LPS can produce inflammation in heart. IL-6 and TNF-α levels increased in ALI rats compared to control group, while pretreatment with p*-*CA (100 mg/kg) significantly inhibited IL-6 and TNF-a level in comparison with LPS-treated rats (*P*<0.01). Zhao *et al*. (2016) demonstrated that p-coumaric acid has an anti-Inflammatory effects in LPS stimulated RAW264.7 cells. When tissues are infected or injured, inflammation occurs, which is a complex process involving immune cells, blood vessels, and molecular mediators (22). The pathological inflammatory procedure leads to activated monocytes, mast cells, macrophages, and lymphocytes, resulting in the producing large amounts of inflammatory mediators including chemokines, and cytokines that damage macromolecules including DNA and the generation of ROS (23).

LPS, a component of the outer membrane of gram-negative bacteria, as an endotoxin is widely used in inflammatory animal models (24). LPS binds to cell membrane receptors (toll-like receptors, or TLRs) of different cells, including endothelial cells and leukocytes, and releases numerous cytokines (25). Cardiac myocytes have also TLRs, especially TLR4. It has been shown that LPS brings down the contractile function of the heart, and since TLR4 is the only LPS receptor, it seems that TLR4 plays a role in the heart function (26, 27).

** *P*<0.01 versus the control group; ## *P*<0.01, # *P*<0.05 versus the LPS group. LPS: Lipopolysaccharide; p-CA: p-Coumaric acid; TNF-α: Tumor necrosis factor alpha; IL-6: Interleukin 6

**Table 1:** ECG records from all groups. Data are expressed as the mean ±SEM (n=8)

**Parameters**	**Control**	**p-CA**	**LPS**	**LPS+p-CA**
**HR (bpm)**	236±3.54	237±5.24	267±1.82**	249±4.34#
**PR ** **interval (S)**	0.053±0.01	0.049±0. 24	0.041±0.01**	0.049±0.02#
**QRS complex (S)**	0.028±0.01	0.026±0.05	0.021±0.02**	0.027±0.01##
**QRS complex (mv)**	0.58±0.02	0.56±0.04	0.37±0.04**	0.55±0.02#
**QT interval (S)**	0.077±0.015	0.071±0.003	0.064±0.001*	0.076±0.001#
**QTc (S)**	0.148±0.003	0.156±0.011	0.139±0.001	0.153±0.003
**RR interval (S)**	0.25±0.005	0.24±0.004	0.2±0.002**	0.24±0.005#

LPS: Lipopolysaccharide; p-CA: p-Coumaric acid; ECG: Electrocardiography. HR: Heart rate

* *P*<0.05,

** *P*<0.01, versus the Control,

# *P*<0.05,

## *P*<0.05 versus the LPS rat.

**Table 2:** Hemodynamic records from all groups. Data are expressed as the mean±SEM (n=8)

**Parameters**	**p-CA**	**Control**	**LPS**	**LPS+p-CA**
**RPP (mmHg/min)**	22037.9±182.5	21314.6±76.2	26364.9±225.2***	21706.5±288.9###
**Perfusion pressure (mmHg)**	61.83±2.36	60.19±1.21	58.34±0.61	63.82±1.68
**+dp/dt (mmHg)**	2406.45±35.97	2479.69±31.57	2281.71±25.35**	2471.4±59.89 #
**-dp/dt (mmHg)**	-2350.25±44.8	-2252.67±31.95	-2403.11±20.06	-2332.01±42.56
**LVDP (mmHg)**	70.47±1.44	69.098±0.68	75.57±1.06 *	70.52±0.79 #
**LVEDP (mmHg)**	6.58±0.28	6.8±0.13	7.98±0.19 *	6.96±0.24 #
**LVSP (mmHg)**	71.94±0.77	68.99±1.1	75.89±3.22	73.59±1.07
**HR (bpm)**	241 ±1.5	247±4.56	280±3.19***	249±2.97###

RPP: rate pressure product; ±dp/dt: Maximal and minimum Rate of Pressure Development; LVDP: left ventricular developed pressure; LVEDP: left ventricular end diastolic pressure; LVESP: Left ventricular end systolic pressure; HR: heart rate; LPS: Lipopolysaccharide; p-CA: p-Coumaric acid

* *P*<0.05,

** *P*<0.01,

*** *P*<0.001 versus the Control,

# *P*<0.05,

### *P*<0.001 versus the LPS

In the current study, it was demonstrated that ALI, induced by LPS, causes myocardial dysfunction in *in vitro* and *in vivo*. Our results showed a significant elevation of the heart rate demonstrated by decreasing of R-R interval. Zhou *et al*. (1991) reported that increased plasma catecholamine concentration is related to the endotoxin, and thereby increases HR (28). QRS, QT interval and QRS voltage showed a significant reduction in LPS group, while QTc interval in LPS group was not significant. These results were also confirmed by Karjalainen *et al*. (29). Pretreatment with p-CA prevented early deterioration of cardio-respiratory parameters in LPS-induced ALI. In patients with sepsis, the cardiovascular system is affected, and many studies have shown that myocardial depression is one of the signs of septic syndromes (30). Determining the direct effects of LPS on the cardiac response that cause alteration in neuro-humoral activity, afterload and preload, is difficult because in response to peripheral hemodynamic changes, the heart is constantly changing (31). In this study, isolated hearts from LPS group showed a significant increase in LVEDP (mmHg) and LVDP (mmHg), and also a decrease in +dp/dt, which is an index of decreased contraction of myocardium. Increasing of oxygen consumption and myocardial work load is due to increase in the RPP (32). However, pretreatment with p-CA in ALI rats recovers these cardiac responses compared to LPS group. Many *in vitro* and animals studies have shown elevated ROS production in the cardiovascular system in response to various stressors and in the failing heart (33). Ion balance is a key element in normal cardiac function, and there is notable data showing that the flux of ion channel and function of ion pump across a cell membrane is altered by ROS in a biological manner in heart tissue (34). ROS causes lipid peroxidation followed by secondary damage to membrane; the mechanism by which this can occur is: suppression of the Ca^2+^ current and alteration in sarcolemma L-type calcium channels (35). A membrane calcium pump whose activity is suppressed by ROS is sarcoplasmic reticulum Ca^2+^ ATPase 2 (SERCA2), which has a critical role in cardiac calcium regulation and acts as a marker of myocardial contractility (36).

In the current study, myocardial function was impaired as demonstrated by increased MDA level. MDA, as a marker of oxidative stress, reflects the effects of reactive oxygen metabolites on the cell damage (37). Endogenous antioxidative factors including SOD, GPx and GSH, had an important function in preventing oxidative stress condition, activated by ROS (38, 39). In agreement with the study of Moura *et al*. (2012) who investigated the effects of *Eutrpe Oeracea Mart* extract on cigarette smoke-induced ALI, in our study there was a significant increase in heart SOD, GPx and GSH and in MDA concentration in LPS group compared to control rats. Treatment by p-CA lowered heart MDA level and antioxidant enzymes compared to the LPS group. This can be a sign of a balance between antioxidants and oxidant elements (40). Prasanna *et al*. (2013) concluded that p-CA could be a critical candidate for protecting the cardiotoxicity induced by sodium arsenite in rats through its antioxidant activity (41). P-CA has been shown to inhibit oxidation of low-density lipoproteins in both *in vitro* and *in vivo* studies (42). In protection against inflammatory tissue injuries, expression of cytoprotective and antioxidative genes follows activation of Nrf2–ARE system (43, 44). In acute inflammation, Nrf2^−/−^ mice compared to wild-type mice showed a significant increase in duration of lung inflammation and susceptibility to pulmonary injury (45). Expressing of cytokines, chemokines, and cell adhesion molecules/receptors were observed at highest levels in Nrf2−/− lungs compared to Nrf2^+/+ ^(46). The pathogenic manifestations of Nrf2 knockout mice were inflammatory lesions in multi-organ, intravascular deposition of immunoglobulin (Ig) complexes and premature death due to rapidly progressing glomerular nephritis (47), in response to pro-inflammatory condition, suggesting that Nrf2 has a critical role in noxious stressors and cellular adaptation.

## Conclusion

In conclusion, this study demonstrated that pretreatment with p-CA attenuated systemic inflammation in ALI model induced by LPS in rats. P-CA reduced oxidative stress, TNF-α, and IL-6 level in heart tissue of LPS group. However, our study did not investigate scavenging role of p-CA in ROS production induced in ALI or whether p-CA can control the synthesis, release, or activity of antioxidant enzymes.
